# Primary Osseous Malignancies of the Spine

**DOI:** 10.3390/diagnostics13101801

**Published:** 2023-05-19

**Authors:** Sisith Ariyaratne, Nathan Jenko, Karthikeyan P. Iyengar, Steven James, Jwalant Mehta, Rajesh Botchu

**Affiliations:** 1 Royal Orthopaedic Hospital, Birmingham B31 2AP, UK; sisith.ariyaratne@nhs.net (S.A.); nathan.jenko@nhs.net (N.J.); stevenjames@nhs.net (S.J.);; 2 Southport and Ormskirk Hospital NHS Trust, Ormskirk L39 2AZ, UK; kartikp31@hotmail.com

**Keywords:** malignant primary vertebral tumours, osteosarcoma, Ewing sarcoma, chondrosarcoma, chordoma, plasmacytoma, lymphoma, angiosarcoma, imaging, MRI, CT

## Abstract

Malignant primary vertebral tumours comprise an uncommon group of primary bone malignancies that can pose a diagnostic and therapeutic challenge. The most frequently encountered malignant primary vertebral tumours include chordoma, chondrosarcoma, Ewing sarcoma and osteosarcoma. These tumours often present with nonspecific symptoms, such as back pain, neurologic deficits and spinal instability, which can be confused for the more commonly encountered mechanical back pain and may delay their diagnosis and treatment. Imaging, including radiography, computed tomography (CT) and magnetic resonance imaging (MRI) is crucial for diagnosis, staging, treatment planning and follow-up. Surgical resection remains the mainstay of treatment for malignant primary vertebral tumours, but adjuvant radiotherapy and chemotherapy may be necessary for achieving complete tumour control depending on the type of tumour. In recent years, advances in imaging techniques and surgical approaches, such as en-bloc resection and spinal reconstruction, have improved the outcomes for patients with malignant primary vertebral tumours. However, the management can be complex due to the anatomy involved and the high morbidity and mortality associated with surgery. The different types of malignant primary vertebral lesions will be discussed in this article with an emphasis on the imaging features.

## 1. Introduction

Malignant vertebral tumours comprise a relatively uncommon group of primary bone tumours arising from the vertebra, affecting both adult and paediatric populations. According to one study, they account for less than 5% of all primary bone tumours [1,2]. The diagnosis of solitary vertebral lesions can be challenging, both due to their rarity as well as the nonspecific nature of presentation, most commonly back pain [3]. The most frequently encountered primary neoplasms in the spine in decreasing order of incidence include chordoma, chondrosarcoma, Ewing sarcoma and osteosarcoma [4].

Early detection and accurate diagnosis are important for effective management. Imaging, particularly computed tomography (CT) and magnetic resonance imaging (MRI) play a crucial role in diagnosing these conditions, and having a sound understanding of the characteristic imaging features of the different types of tumours is paramount. Treatment options depend on the extent and type of the tumour, but surgical resection remains the mainstay of management in localised cases. En-bloc resection in the spine may be performed for surgical resection of these tumours and has been proven to lengthen disease-free survival and decrease the likelihood of local recurrence [5,6]. Management, however, is also challenging as surgical resection of these tumours is associated with high morbidity and mortality. Adjuvant methods of treatment such as chemotherapy, radiotherapy, proton beam therapy, percutaneous techniques such as ablation and targeted therapies are also used. The management is further complicated due to the limitations of using local adjuvants around the spine due to the risk of damage to critical structures, in particular the spinal cord [7,8].

The aim of this review article is to discuss the different types of primary malignant tumours of the vertebral column, with emphasis on the approach to imaging and distinguishing imaging features. Discussion of secondary tumours such as metastases and multiple myeloma, which commonly affect the vertebrae, as well as benign lesions and lesions of infective aetiology will be excluded from this discussion. 

## 2. Imaging Modalities and Techniques

While conventional radiography may be used as a preliminary screening tool, it is of limited value in characterising primary neoplastic lesions of the spine due to the complex anatomy of the spine [2]. CT and MRI are the main imaging modalities used.

MRI remains the preferred imaging modality [2] and provides valuable diagnostic information in the assessment of these lesions. The imaging can be generally performed in either a 1.5 or 3 Tesla MRI, depending on availability. In addition to standard sequences, i.e., T1, T2, the inclusion of a sagittal STIR (short tau inversion recovery) fat-suppressed T2 image is useful when assessing for marrow infiltration or oedema, which, in some instances, may be subtle in standard T1 sequences and occult on T2, and has high positive and negative predictive values in assessing for vertebral marrow abnormalities, 99.3% and 95.95, respectively [1]. Post gadolinium contrast sequences, diffusion weighted sequences and chemical shift imaging may also be used [2,9]. 

In the absence of marrow oedema or signal change on the STIR sequences, the addition of post-contrast sequences usually does not add any diagnostic value, but post-gadolinium T1 sequences can be helpful in the presence of marrow signal change. Diffusion weighted imaging can be of value in distinguishing malignant lesions from benign entities, such as in cases of pathological vertebral collapse, as it provides an indication of the lesion cellularity, with cellular lesions demonstrating increased diffusion restriction [9,10,11]. However, it can be poor at distinguishing between infection and malignancy as both these entities can show increased diffusion restriction. The sequences should be reviewed in conjunction with standard sequences such as T1 and T2, as these sequences lack anatomical detail. In certain instances, it can yield falsely negative results, such as when assessing lesions treated with radiotherapy, sclerotic lesions or heavily necrotic lesions, which can be a diagnostic pitfall [1]. 

Chemical shift imaging with in-phase and opposed-phase sequences is a useful adjunct in distinguishing benign and malignant lesions. A lesion demonstrating a signal intensity drop of >20% on the opposed-phase imaging, suggesting the presence of significant intralesional fat, favours a benign diagnosis and, conversely, a lesion demonstrating <20% signal loss on opposed-phase imaging is more likely to be malignant. A cut-off value of 35% has a sensitivity of 95%, specificity of 100%, positive predictive value of 100% and negative predictive value of 95.2% in diagnosing malignancy [9]. However, again, one must take caution in interpreting this due to the presence of false negatives, as certain malignant lesions containing fat such as post-radiotherapy lesions or myeloma can cause a significant drop in signal intensity in opposed-phase imaging [1,12]. 

A 3D T1 volumetric interpolated breath-hold examination (VIBE) sequence is also increasingly used. This has the benefit of increased spatial resolution over standard MRI sequences and can be used to generate high-quality multiplanar reformats from the original dataset. VIBE sequences are characterised by lower fluid signal characteristics compared to standard T1 weighted images but are otherwise similar to standard T1 weighted images. Furthermore, the sequences can be inverted and windowed to generate high-resolution images resembling the attenuation pattern of a CT, in some instances negating the need for additional CT [13].

CT is particularly useful in assessing the bone anatomy, degree of osseous destruction and assessing for the presence of matrix calcification or ossification, which can be seen with certain tumours such as osteosarcoma [2]. Measurement of Hounsfield units (HU) in certain lesions is a helpful and reliable method of distinguishing benign sclerotic lesions such as an enostosis from malignant entities, including osteoblastic metastases. Using a mean attenuation cut-off of 885 HU can distinguish benign lesions such as enostosis (which tend to have greater mean HU values than 885) from a malignant entity, with a 95% sensitivity and 96% specificity rate [12]. 

CT is also a valuable tool in planning for and guiding biopsies of these lesions. CT-guided biopsies are relatively safe and invaluable in providing a histological diagnosis prior to definitive management. The biopsy approach should be planned with the input of the surgical team and considerations for future excision where appropriate to minimise the risk of tumour seeding [14]. 

Angiographic imaging, either CT or MRI, may also be useful, particularly when characterising lesions in the cervical spine, where assessing the relationship to the neck vessels is important when planning surgical management and also for characterising highly vascular tumours such as angiosarcoma [2]. Radionuclide (bone) scans and PET scans are mainly utilised for staging and surveillance and for assessing multiple lesions and distant sites of disease [2] but tend to have a limited role in characterising solitary vertebral lesions. 

Future research should continue to explore novel imaging techniques and refine existing modalities to enhance diagnostic accuracy, monitor treatment efficacy and improve patient care. Collaboration between radiologists, oncologists, pathologists and surgeons is paramount for the multidisciplinary management of primary malignant tumours of the spine. By implementing advanced imaging techniques, including additional advanced MRI sequences such as those discussed above, healthcare professionals can make significant progress in the early detection and effective treatment of these often aggressive and debilitating neoplasms.

## 3. Types of Lesions

The various types of primary malignant vertebral tumours can be classified based on their tissue of origin and type of cells present (Table 1). The types of tumours are discussed in greater detail below. 

### 3.1. Osteosarcoma

Spinal osteosarcomas are rare and only account for approximately 4% of all primary malignant vertebral tumours and 5% of all primary osteosarcomas. The majority (up to 33%) are seen in the thoracic and lumbar spine [1]. Approximately 80% arise eccentrically in the neural arch and extend into the vertebral body. The lumbar spine is commonly involved (33%), followed by the sacrum and thoracic spine. Patients present with back pain; often insidious and neurological symptoms can occur due to tumour invasion resulting in cord or nerve root compression. Incidence peaks in the fourth decade of life.

**Figure 1:**
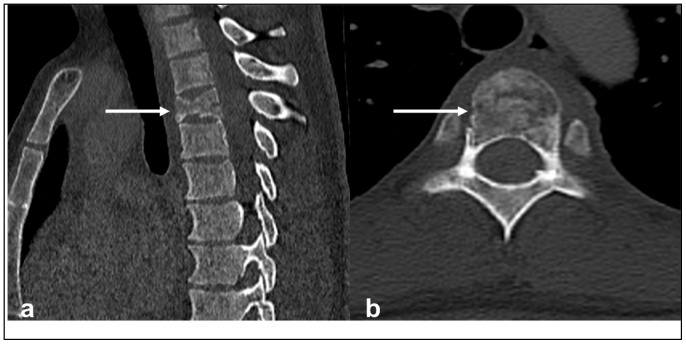
Sagittal (**a**) and axial (**b**) CTs demonstrating primary osteosarcoma in the upper thoracic spine (white arrows). Note the osteoid matrix, characterised by an area of sclerosis, as well as lytic areas. A pathological fracture is also present.

**Figure 2:**
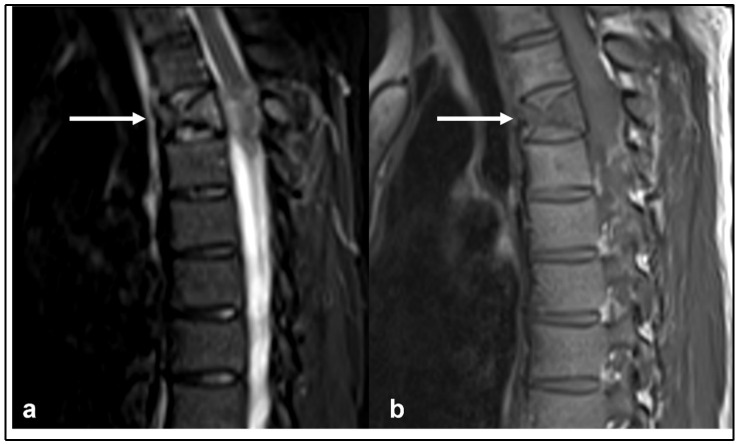

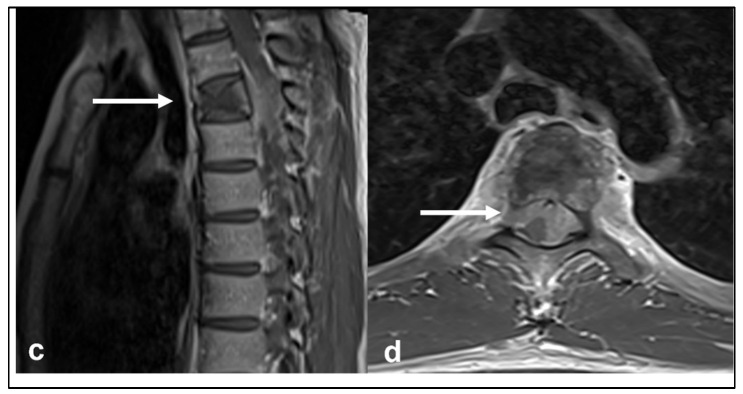
Sagittal STIR (**a**) and T1 (**b**) sequences and sagittal (**c**) and axial (**d**) post-gadolinium contrast sequences showing the osteosarcoma of the upper thoracic spine (white arrows). The large extra-osseous soft tissue component extending into the canal and relationship to adjacent anatomical structures is well demonstrated.

On imaging, the lesions are typically osteoblastic with a dense osteoid matrix. Less commonly, they can present as purely lytic lesions. Cortical breakthrough with an extra-osseous soft tissue component is a common finding. CT is a useful imaging modality, providing a detailed assessment of the osteoid matrix and extra-osseous soft tissue components. The sclerotic and lytic areas can be easily assessed on CT. Intravenous contrast should be avoided when possible as this limits the visualisation of the osteoid matrix, which is an important distinguishing feature of osteosarcoma [14] (Figure 1a,b). MRI is particularly useful in assessing the extent of the extra-osseous extent of the tumour although the matrix is poorly demonstrated on MRI (Figure 2a–d). Telangiectatic osteosarcoma, a rare and particularly aggressive form of osteosarcoma, can have fluid–fluid levels on MRI, which can be mistaken for an aneurysmal bone cyst [2]. The tumours typically demonstrate uptake in all three phases of a bone scan and are typically used for staging and assessing for skip lesions [14].

The mainstay of treatment includes surgical resection when feasible, ideally an en-bloc resection [5,6], as well as radiotherapy and chemotherapy [1]. 

### 3.2. Ewing Sarcoma

Ewing sarcoma commonly occurs in the younger population, with a peak incidence between the ages of 10 and 30 years. It is the most common primary malignant bone tumour of the spine in children and accounts for 3.5–10% of cases of Ewing sarcoma in children [1,15]. The sacrum is most commonly involved (approximately 55% of cases), followed by the lumbar spine (25%), thoracic spine (10%) and cervical spine (approximately 3%) [1]. In addition to Ewing sarcoma of the spine typically arising in the vertebral bodies, a very rare form of extra-skeletal Ewing sarcoma also occurs in the extradural location within the spinal canal [1,16].

The lesions typically arise in the neural arch but often extend into the vertebral body, and isolated vertebral body or neural arch involvement is rare. Extra-osseous soft tissue component is usually seen. The majority of tumours, approximately 93% according to one study, are lytic and result in vertebral collapse, and less commonly sclerotic lesions are also seen [1,15]. 

**Figure 3:**
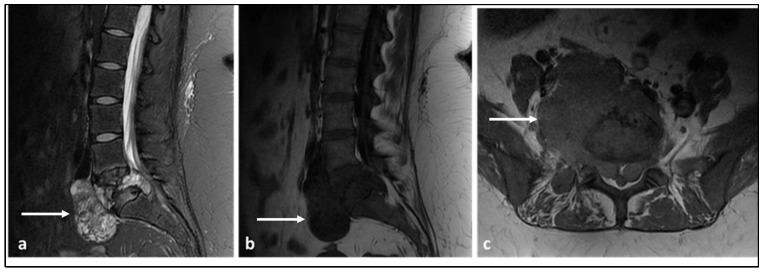
Sagittal STIR (**a**), sagittal T1 (**b**) and axial T1 (**c**) MRI sequences demonstrating a primary vertebral Ewing sarcoma centred in the L5 vertebral body (white arrows). Note the involvement of both pedicles and large extra-osseous soft tissue component extending to the anterolateral paravertebral regions, epidural space and both foramina, the latter causing compression of the thecal sac and exiting L5 nerve roots. The lesion demonstrates the signal characteristics typically seen, with low to intermediate T1 and intermediate to high signal on the fluid-sensitive sequence.

MRI is the mainstay imaging modality. There are no imaging features that are specific for Ewing sarcoma and the lesions often appear as a low to intermediate signal T1 and intermediate to high signal T2 lesion. When sclerosis is present, these areas will result in a low T1 and T2 signal [1]. Paravertebral soft tissue components, which are present in most cases (Figure 3a–c), demonstrate post-contrast enhancement and may show increased restriction of diffusion due to high cellularity. The soft tissue components tend to extend into the epidural space and foramina, resulting in cord, cauda equina and/or nerve root compression. In CT, the lesions have a typically lytic or permeative appearance and, less commonly, sclerotic hyperdense foci can be seen [17]. While substantial soft tissue components can be visualised, particularly with contrast, the extent of involvement is poorly characterised in CT. 

### 3.3. Chondrosarcoma

The gradual but widespread increase in MR imaging has resulted in more frequent diagnoses of chondrosarcomas and even more so of borderline malignant lesions, now referred to as atypical cartilaginous tumours (ACTs) [18]. Nevertheless, chondrosarcoma of the spine remains rare, with a search of the US National Cancer Institute’s databases identifying only 973 cases between 1973 and 2012 [19], but, notably, 61.2% of cases were diagnosed after 2000. The mean age at diagnosis was 51.6 years with a slight 3:2 predilection for males [20]. Although a slowly progressive disease, recurrence is common and the median survival after diagnosis is 6.9 years [19].

Chondrosarcomas have a mild predilection for the thoracic spine, followed by the lumbar and cervical spine [21]. Primary lesions occur more frequently in the vertebral body, while secondary chondrosarcoma due to the degeneration of chondral lesions is more frequent in the posterior elements [22]. The latter can also be due to underlying osteochondroma [23].

**Figure 4:**
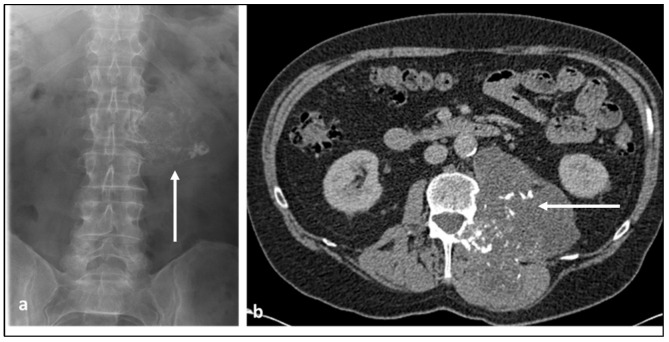
AP radiograph of the lumbar spine (**a**) and axial CT of the abdomen (**b**) demonstrating a chondrosarcoma arising from the left lateral aspect of the L2 vertebra, involving the body, left transverse process and pedicle (white arrows). Large soft tissue component with internal “rings and arcs” type calcification, in keeping with a chondroid matrix, is a characteristic feature.

**Figure 5:**
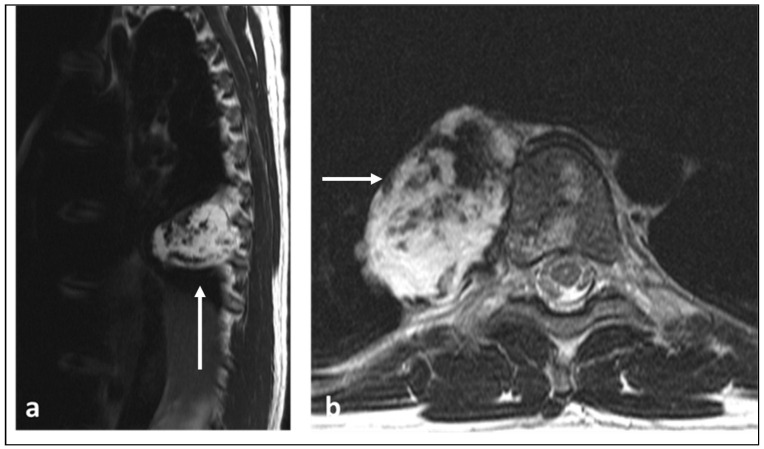

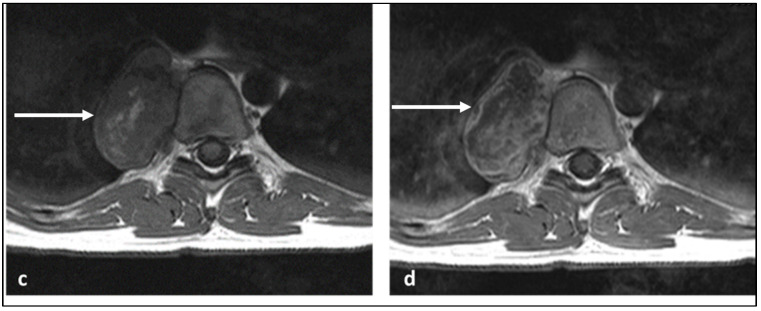
Sagittal T2 (**a**), axial T2 (**b**) and axial T1 pre- (**c**) and post-contrast (**d**) sequences showing a chondrosarcoma of a mid-thoracic vertebra (white arrows). Note the typical features with predominant high T2 signal, low T1 and T2 areas representing calcification (chondroid matrix) and peripheral and septal enhancement. There is also a large extra-osseous soft tissue component, which is commonly seen.

These neoplasms are primarily comprised of hyaline cartilage, which is organised in a lobular pattern. The ossification surrounding the hyaline cartilage is responsible for the well-known “rings-and-arcs” appearance in CT and X-ray (Figure 4a,b). The cartilage has a high water content, which, along with myxoid contents, is responsible for the very high T2 signal demonstrated in MRI (Figure 5a,b) and low density in CT. Higher-grade lesions tend to have larger noncalcified areas in keeping with the more rapid production of the chondroid matrix [24]. Peripheral and septal enhancement can be seen in post-contrast imaging in MRI [1] (Figure 5c,d). The defining feature of chondrosarcoma compared to benign cartilaginous tumours is the destruction of adjacent trabecular bone [24], with larger tumours often extending into a large soft tissue mass beyond the osseous spine.

The mainstay of treatment is surgery, with adjuvant radiotherapy only being considered in more aggressive (pathology grade III) diseases. Higher pathological grades and the presence of metastasis at diagnosis are also poor prognostic factors [20]. The most common site of metastasis is the lungs, with calcification within pulmonary nodules making metastasis more likely [25].

Careful delineation of the disease using CT is essential for surgical planning—multiple grading systems have been proposed to describe the size, position and resectability of the tumour, with the Weinstein, Boriani, Biagini (WBB) clock-face classification being most widely used [26]. Total en-bloc resection is independently associated with a good prognosis but is often not achievable due to anatomical constraints. Hence, local recurrence rates are significant with reports ranging from 27% [27] to 42% [20].

### 3.4. Chordoma

Chordomas are tumours that grow slowly and invade local tissues, with distant metastases occurring only in rare cases and usually in the advanced stages of the disease. Despite being classified as indolent tumours, chordomas pose a significant risk of recurrent growth in the local area. As a result, lifelong monitoring post-surgical resection and radiotherapy is required, ideally using MRI. Peak incidence is during the fourth to seventh decades of life [1,28].

They originate from the notochord, which is the mesodermal component of the embryo, which ultimately becomes the nucleus pulposus; hence, these tumours are seen in the midline and arise from the vertebral bodies, with the neural arch being typically spared. They are most commonly seen in the sacrum (over 50%) and the remainder occur in the vertebral bodies of the mobile spine, most commonly C2, followed by the lumbar and thoracic spine. They are the most common primary tumour of the sacrum [28].

**Figure 6:**
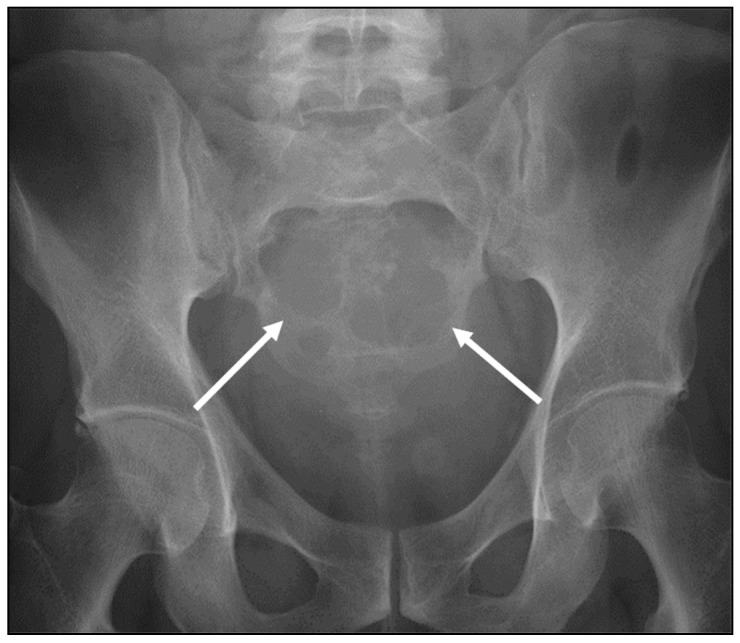
AP pelvic radiograph of a patient with sacral chordoma demonstrating the osseous destruction of the sacrum (white arrows).

**Figure 7:**
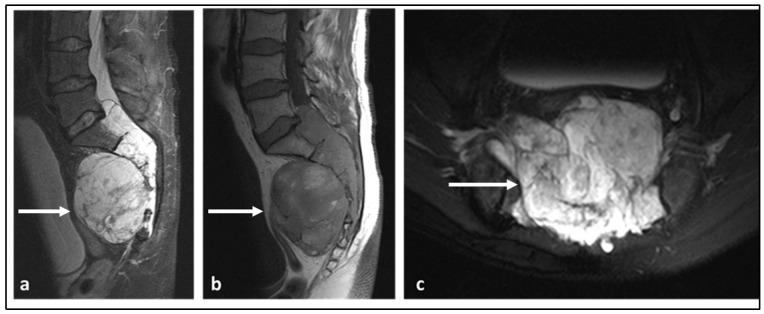
Sagittal STIR (**a**), sagittal T1 (**b**) and axial STIR (**c**) sequences highlighting a large sacral chordoma (white arrows). Note the locally destructive nature of the lesion with large extra-osseous soft tissue component. The lesion shows predominant high fluid signal in keeping with myxoid content and foci of high T1 signal indicative of haemorrhage and/or mucinous content.

On imaging, a chordoma typically presents as a large destructive midline mass arising from either the sacrum or a vertebral body, with a secondary soft extra-osseous extension. The lesions have a high myxoid content, which manifests as areas of low attenuation and destruction radiographs and CT *(*Figure 6) and high signal in fluid-sensitive sequences in MRI. Areas of punctate calcification and haemorrhage within the lesion are also common. While high T2 signal intensity is not a definitive characteristic and is commonly seen in most spinal tumours, the presence of a sacral mass with a lobulated appearance accompanied by haemorrhagic and calcified regions should raise suspicion of a chordoma (Figure 7a–c). The lesions typically have a lower T1 signal compared to skeletal muscle, with the exception of the haemorrhagic and mucinous foci within the tumour, which may be T1 hyperintense. Vertebral collapse is also a feature [1,29].

As with most primary spinal malignant tumours, the best survival outcomes are achieved with en-bloc resection with clear margins. If this cannot be achieved, piecemeal resection or debulking are also viable options. Due to the high recurrence rate, radiation therapy is usually recommended after surgical resection. Chordomas tend to be radioresistant and hence require high-dose radiation therapy. Due to their slow growth, they usually do not respond well to conventional chemotherapy [28]. Proton beam therapy is commonly used and has been associated with significantly improved tumour control and survival outcomes [30]. In recent years, carbon fibre implants have been used for surgical stabilisation, as these have been associated with superior target coverage and improved outcomes when proton beam therapy is used [31].

### 3.5. Lymphoma

Primary bone lymphoma only occurs rarely in the spine, with most occurring as a metastasis from other sites. The largest published case series includes 237 cases of primary bone lymphoma with only 11 cases occurring in the vertebral column1 [32]. Non-Hodgkin’s lymphoma is much more common than Hodgkin’s bone lymphoma; diffuse large cell B-cell lymphoma (DLBCL) is the most common subtype [33].

**Figure 8:**
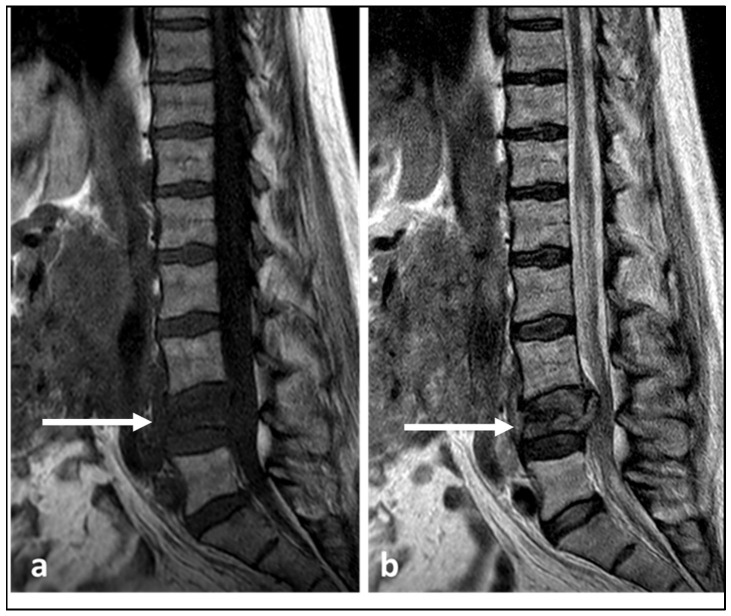
Sagittal T1 (**a**) and T2 (**b**) sequences demonstrating a primary lymphoma of the L4 vertebra with typical low T1 and T2 signal within the lesion (white arrows). There is also partial vertebral collapse.

The defining feature of bone lymphoma is permeative infiltration of the bone marrow without cortical destruction. In the majority of cases, an adjacent soft tissue mass is present [32,34]. The soft tissue mass is sometimes visible on plain film, but CT and particularly MRI enable accurate delineation of the soft tissue mass. Most commonly, the T1 and T2 signal is reduced (Figure 8a,b), but nodular sclerosing lymphoma can be T2 hyperintense [35]. Typically, both the vertebral body and arch are involved and there can be vertebral collapse. Extra-osseous extension into the paravertebral soft tissues and epidural space is also common [1].

Nuclear medicine techniques are also useful to determine the spread of the disease; lymphoma demonstrates avid uptake on both bone scintigraphy and 18-F fluorodeoxyglucose (18-F FDG) based studies [33]. Additionally, functional imaging with 18-F FDG is particularly useful in the assessment of treatment response in line with the Lugano classification.

Due to the rarity of primary bone lymphoma, only limited data are available on treatment outcomes. The largest case series reported contained 83 patients [36], 46 of which received the gold-standard treatment of radiotherapy and chemotherapy. In this group, the five-year survival was high at 90%.

### 3.6. Plasmacytoma

Plasmacytoma is a solitary form of multiple myeloma. While commonly seen in the middle-aged to elderly population, the age of presentation on average tends to be about 10 years younger than for multiple myeloma. It can remain localised for many years before progressing to generalised multiple myeloma [1].

**Figure 9:**
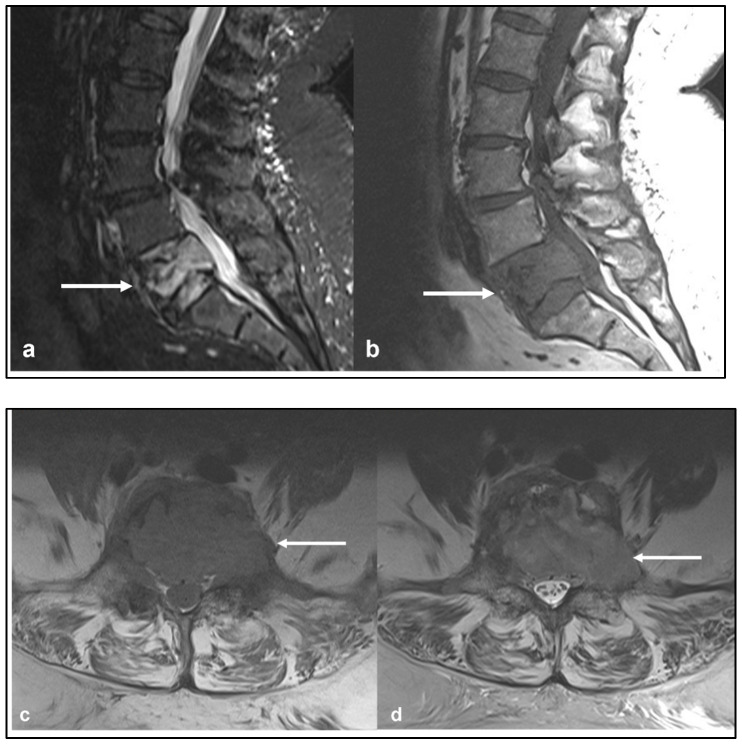
Sagittal STIR (**a**), T1 (**b**), axial T1 (**c**) and T2 (**d**) sequences demonstrating a solitary plasmacytoma of L5 vertebral body (white arrows). While there are no specific characteristic features, low T1 and high fluid signal with vertebral collapse are present.

In imaging, plasmacytomas are typically lytic and destructive and hence vertebral collapse can be seen. They have a low to intermediate signal on T1 sequences in MRI and an increased signal in fluid-sensitive sequences (Figure 9a–d) and enhancement is seen with contrast. Extra-osseous soft tissue component is also a common finding. The lesions typically involve the vertebral body and extend to the pedicles. The presence of multiple trabeculae within the lesion, with a characteristic soap bubble appearance, is a distinguishing feature [2]. Following diagnosis, either a skeletal survey or an MRI of the whole spine should be considered to exclude additional lesions. 

Histological grade often dictates management and a moderate-dose radiotherapy combined with surgery is occasionally suggested for optimal treatment with sufficient local control. Patients with a high-grade histology may benefit from adjuvant chemotherapy [37].

### 3.7. Angiosarcoma and Epithelioid Haemangioendothelioma (EHE)

Angiosarcoma is an aggressive high-grade vascular neoplasm, which can either arise from vascular or lymphatic tissue. Similarly, EHE is also an aggressive vascular tumour, comprised of cords of eosinophilic cells and anastomosing vascular channels. These tumours are very rare and even more so in the vertebrae, accounting for less than 1% of primary vertebral tumours [1,38]. Peak incidence is during the second to fourth decades of life. Angiosarcoma has a recognised association with prior radiotherapy to the region and also lymphoedema [1].

They are typically aggressive and appear as lytic lesions in imaging. Characteristic features in imaging include the presence of high-flow vascular channels, which can be seen in contrast-enhanced CT and also MRI in the form of serpiginous flow voids. They have a predilection for the vertebral body [1,38].

Due to the highly vascular nature, angiography and embolisation may be needed prior to surgical resection [38].

## 4. Conclusions

Various imaging modalities, particularly CT and MRI, play a crucial role in detecting and characterising primary osseous malignancies of the spine (Table 2), as well as in early and accurate diagnosis, which ultimately impacts the prognosis, management and patient outcomes. MRI has emerged as the gold standard in imaging of these tumours, offering superior soft tissue resolution and the ability to assess the tumour extent and involvement of adjacent structures. Advanced MRI techniques, such as DWI and chemical shift imaging, have further improved our understanding of these tumours and response to treatment. Conversely, CT provides excellent bony detail and aids in surgical planning, while PET has shown promise in differentiating between benign and malignant lesions, as well as in assessing response to therapy. Understanding the imaging features of the various tumours can guide radiologists and clinicians in formulating differential diagnoses, ultimately leading to prompt and appropriate management strategies.

## Figures and Tables

**Table 1:** Classification and types of primary malignant vertebral tumours.

Classification	Tumour
Osteogenic	Osteosarcoma
Round cell tumours	Ewing sarcoma
Chondrogenic	Chondrosarcoma
Notochordal origin	Chordoma
Haematopoietic	Lymphoma Plasmacytoma
Vascular	Angiosarcoma Epithelioid Haemangioendothelioma

**Table 2:** A summary of pertinent CT and MRI features of the aforementioned primary malignant vertebral tumours.

Tumour	CT Features	MRI Features
Osteosarcoma	Osteoid matrix with sclerotic and lytic areas. Contrast can obscure osteoid matrix.	Extra-osseous soft tissue component. Low T2 signal foci corresponding to mineralisation. Secondary ABC change with fluid–fluid levels (this can be seen with telangiectatic osteosarcoma also).
Ewing sarcoma	Lytic lesion. Vertebral collapse.	Low to intermediate signal T1 and intermediate to high signal T2 lesion. Enhancing extra-osseous soft tissue component.
Chondrosarcoma	Lytic lesion. ‘Rings and arcs’ calcification characteristic of chondroid matrix.	High T2 signal due to cartilage and myxoid content. Peripheral and septal enhancement. Enhancing extra-osseous soft tissue component.
Chordoma	Low attenuation lytic lesion.	Loculated high T2 signal mass, usually with extra-osseous soft tissue component. High T1 signal due to haemorrhagic and calcified components.
Lymphoma	Permeative bone loss without overt destruction.	T1 and T2 signal is reduced, but nodular sclerosing lymphoma can be T2 hyperintense. Extra-osseous soft tissue component.
Plasmacytoma	Lytic and destructive, vertebral collapse. Soap bubble appearance.	Low to intermediate signal on T1 and increased signal on fluid-sensitive sequences and enhancement post-contrast. Extra-osseous soft tissue component. Multiple trabeculae within the lesion, with a characteristic soap bubble appearance.
Angiosarcoma and Epithelioid Haemangioendothelioma	Lytic lesion.	High flow serpiginous vascular channels.

## Data Availability

Not applicable.
